# Efficacy of monthly treatment with oral fluralaner (Bravecto^®^ 1-Month) against *Tunga penetrans* in dogs in Brazil: a randomized, double-blind, controlled field study

**DOI:** 10.1186/s13071-024-06272-y

**Published:** 2024-04-29

**Authors:** Katharine Costa dos Santos, Paula Elisa Brandão Guedes, George Rêgo Albuquerque, Anderson Vieira de Jesus, Anaiá da Paixão Sevá, Joana Thaisa Santos de Oliveira, Jamille Bispo de Carvalho Teixeira, Thammy Vieira Bitar, Tatiani Vitor Harvey, Sofia Nadir Sanches Ramos, Francisco Bonomi Barufi, Fernando de Almeida Borges, Renata Santiago Alberto Carlos

**Affiliations:** 1 Department of Agricultural and Environmental Sciences, Postgraduate Program in Animal Science, UESC, State University of Santa Cruz, Rod. Jorge Amado Km 16—Salobrinho, Ilhéus, Bahia 45662-900 Brazil; 2 Texas A&M University, College Station, 400 Bizzell St., TX 77843 TX USA; 3 MSD, Merck & Co Animal Health, Avenida Doutor Chucri Zaidan, 296, 12º Andar, São Paulo, 04583-110 Brazil; 4 Faculty of Veterinary Medicine and Zootechnics, UFMS, Federal University of Mato Grosso Do Sul, Campo Grande, Mato Grosso Do Sul Brazil

**Keywords:** Canine tungiasis, Ectoparasitosis, Treatment, Rural communities, Zoonosis

## Abstract

**Background:**

Tungiasis is a neglected tropical disease caused by the adult female sand flea (*Tunga penetrans*). Dogs are considered important reservoirs of *T. penetrans* in Brazil. The aim of this study was to determine the monthly insecticidal efficacy of a single oral administration of fluralaner at a dose of 10–18 mg/kg (Bravecto^®^ 1-Month, also registered as Defenza^®^ in some countries; MSD Animal Health) in dogs naturally infested with *T. penetrans*.

**Methods:**

This clinical trial was conducted in a rural community located in Ilhéus, Bahia, Brazil. A total of 64 dogs were selected and distributed in a completely randomized design between a treated group (TG) that received one single dose of Bravecto^®^ 1-Month (Defenza^®^) and a negative control group (CG) that received no treatment. Each group was composed of 32 dogs. The evaluations took place on days 0, 7 ± 2, 14 ± 2, 21 ± 2, 28 ± 2, 35 ± 2, and 42 ± 2 post treatment, in which the dogs were inspected to evaluate the infestation stage and classify lesions associated with tungiasis. The primary efficacy was determined from the percentage of treated dogs free of fleas (stage II and III lesions) after administration of the formulation at each evaluation time. Secondary efficacy was based on the number of active lesions (stages II and III) in each group at each evaluation time. The clinical condition of the animals was defined based on the Severity Score for Acute Dog Tungiasis (SCADT), which is related to the number and severity of lesions.

**Results:**

The primary efficacy of the product was greater than 95.0% from days 7 to 21 and reached 100.0% between days 28 and 42, with a significant association between treatment and infestation decline (*P* < 0.025) between days 7 and 42. Secondary drug efficacy was greater than 99.9% from days 7 to 21, reaching 100.0% between days 28 and 42 (*P* < 0.05). The treated dogs also scored lower on the SCADT than the control animals did during the entire clinical evaluation period (*P* < 0.05).

**Conclusions:**

A single administration of Bravecto^®^ 1-Month (Defenza^®^) was effective in eliminating *Tunga penetrans* infestations, as well as in preventing parasitism for at least 42 days after treatment.

**Graphical Abstract:**

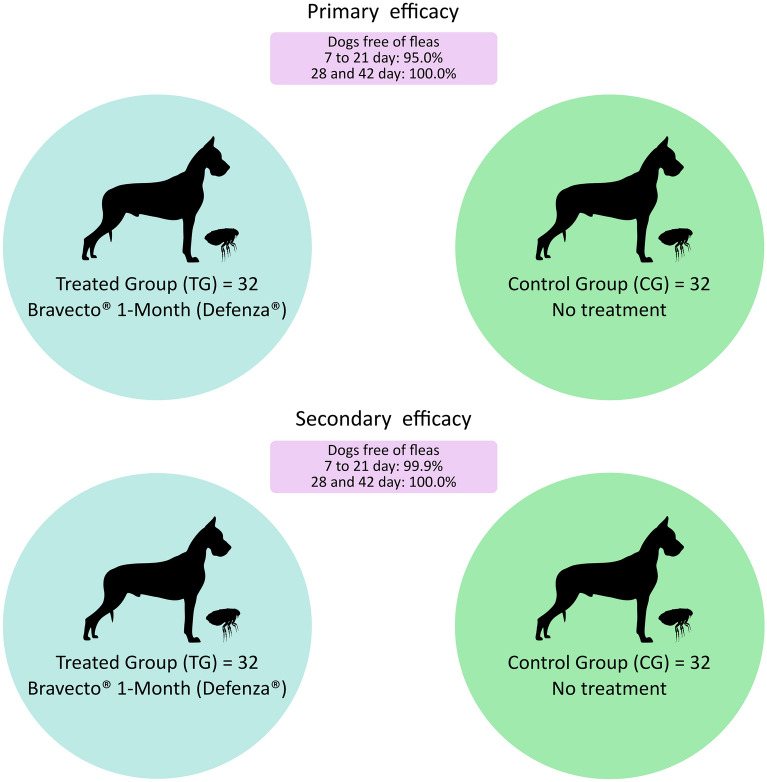

## Background

Tungiasis is a neglected tropical disease (NTD) [1, 2], caused by the adult female sand flea (*Tunga penetrans*) [3]. Dogs can also be infested by *T. penetrans* [4, 5]. Direct contact with contaminated soil predisposes the host to penetration, which usually occurs in the paw regions. After penetration, fleas hypertrophize their neosomes, mature, and eliminate their eggs, remaining in situ until their death, which occurs 4–6 weeks after penetration [4–7]. In most cases, the hosts have a high parasitic load [8], predisposing them to complications such as secondary bacterial infections [9, 10] that progress to deformity and/or loss of digit(s), self-mutilation, septicemia, and death in dogs [11].

In Brazil, dogs are the most affected domestic reservoir and are considered the main disseminators of these ectoparasites [8–14]. Moreover, close contact with this species is considered one of the main risk factors for *T. penetrans* infestation in humans [15]. The high prevalence of tungiasis in humans and animals is associated with low socioeconomic indicators in endemic communities [4, 7], especially in fishing villages [8, 10], indigenous communities [5], slums [9], and rural areas [8].

The participation of dogs in the dynamics of the disease is evident in endemic locations, as these animals have a wide circulation area and cover large perimeters, spreading flea eggs in the environment and facilitating the establishment of the parasite's biological cycle [15, 16]. Environmental contamination caused by infested animals can be prevented by controlling the fleas in the host. This would cause a decrease in reservoirs and a subsequent reduction in prevalence rates in humans and animals [17, 18].

In both humans and animals, the distal regions of the limbs are most affected by infestations because of frequent contact with contaminated soil [14]. However, ectopic lesions on the lips, muzzles, elbows, mammary glands, and genitals are not rare [11]. Infested dogs commonly harbor tens or hundreds of sand fleas, which favor the formation of lesion clusters and aggravate the disease [15, 17–19].

Currently, only a few treatments are available for canine tungiasis. The combination of 10.0% imidacloprid and 50.0% permethrin (Advantix^®^) has approximately 80.0% efficacy in treating and preventing *T. penetrans* re-infestations for 14 days; however, the efficacy decreases significantly to approximately 30.0% in the following week [20]. In addition, subcutaneous treatment with ivermectin (Ivomec 1.0%) healed the lesions in infested dogs 1 month after administration [21]. However, none of these treatments are acceptable for pesticide use according to the European Medicines Agency, which defines the minimum efficacy of a standard product as 95.0% for adult fleas and at least 90.0% for emerging adult fleas [22]. Recently, treatments with a single dose of 25–56 mg/kg fluralaner (Bravecto^®^), a molecule from the isoxazoline group, showed high and persistent systemic insecticidal efficacy against *T. penetrans* in dogs from day 7 to 120 post treatment. The number of live fleas in treated dogs was reduced by > 90.0% on day 7, > 95.0% between days 14 and 90, and 100.0% from day 21 to 60 [23].

However, considering the cost and feasibility of administering fluralaner with quarterly effectiveness, administration of the same molecule at a shortened duration would enable its use in cases of low environmental pressure or low resources, as is the case in deprived communities. Moreover, the treatment may serve as a tool for public managers of future programs to control this disease and a feasible option for use in dogs that travel to endemic areas for a short period of time, ensuring the protection of these animals [23].

This study presents results of a clinical study on the Bravecto^®^ 1-Month (also registered as Defenza^®^ in some countries; MSD Animal Health) formulation to control and prevent canine tungiasis. The study sought to determine the efficacy of the formulation against *T. penetrans* in naturally infested dogs, treated once orally, as well as the impact of the treatment on the clinical conditions of the treated animals. Additionally, acute clinical signs of tungiasis during the study period are listed.

## Methods

### Study area and population

The study was carried out in the semi-rural community Vila Juerana, Ilhéus, Bahia, Brazil (14° 47ʹ 00" S, 39° 03ʹ 00" W). The village is located at a mangrove and beach region that is considered as a tourist area of the Costa do Cacau region, with an annual temperature variation of 22–25 ℃, regular rainfall distributed throughout the year, and a humid tropical climate [24]. Most of the village community have precarious sanitary and social conditions. The streets are unpaved and consist of sandy and clay soil, and some buildings are unfinished without concrete floors [8, 25]. Moreover, the study area had previously shown a high year-round prevalence of tungiasis in dogs [8, 16, 23–26].

Prior to the start of the study, dog owners were informed about the research objectives and methodology and provided written consent for the inclusion of their dogs.

As inclusion criteria, dogs were ≥ 8 weeks in age, had body weights ≥ 2.5 kg, had adequate temperament to allow clinical and parasitological evaluations, and presented at least one live flea at stage II or III lesion in the Fortaleza classification [3]. The selected dogs underwent clinical evaluation, and only animals with no clinical signs other than tungiasis were enrolled in the study, regardless of sex or breed. Dogs treated with fluralaner less than 90 days before day 0 or with other ectoparasiticides with short acting activity within 14 days before day 0 (isoxazolines, amitraz, fipronil, macrocyclic lactones, or pyrethroids), as well as pregnant or lactating female dogs, were not allowed to participate in the trial. Throughout the study period, the dogs remained under the care of their owners and maintained their usual routines.

### Study design

This field study was randomized, negatively controlled, and double-masked. On day 0, 64 dogs were randomly distributed (using a prior computer-generated list) into one of two experimental groups, each composed of 32 dogs. The dogs were implanted with numerically coded microchips for individual identification. The clinical history of each dog was recorded, and each dog underwent a complete physical examination, including ectoparasite assessment.

Study activities were distributed between the two veterinary teams. One team assigned the animals to groups, administered the treatments, and did not participate in the clinical evaluations. The other team performed the clinical evaluations and inspections of the dogs and was blinded to the treatment assignments. Dogs in the treatment group (TG) received a single oral fluralaner dose (Bravecto^®^ 1-Month/Defenza^®^) at the approved dose rate of 10–18 mg fluralaner/kg body weight, according to label instructions, on day 0. The dogs in the control group (CG) remained untreated.

All animals were treated on the same day, received wet commercial dog food, and the owners were not informed of the group to which their dogs were assigned. During the study, if necessary, dogs were treated for any observed secondary complications of tungiasis as the treatment presented no insecticide activity or any other routine health needs. The administration of any other parasiticidal drug or product with insecticidal activity was prohibited. In the end of the study, all 64 dogs were treated with a dose of 25–56 mg/kg of fluralaner (Bravecto^®^).

### Clinical evaluation, skin inspection, and lesion documentation

Dogs were evaluated weekly with seven scheduled examinations for each dog including days 0 (enrolment and treatment), 7 ± 2, 14 ± 2, 21 ± 2, 28 ± 2, 35 ± 2, and 43 ± 2.

At each visit, the dogs underwent a general physical examination and detailed skin inspection. The entire body of the animal was examined, with special attention paid to the paws, limbs, tail, mammary glands, abdomen, testicles, and nose. Before the examination, the dogs’ paws were cleaned using water and a brush to improve the detection of all lesion stages. The identified lesions were counted and staged according to the Fortaleza classification [3].

Severity Score for Acute Dog Tungiasis (SCADT) were also assigned to each dog to record the severity of tungiasis lesions throughout the study. Each clinical sign was scored for each affected area and the results were added to obtain the SCADT (Table 1). The maximum possible score for this classification was 27. If the SCADT score exceeded 22, the dog would be treated by surgical removal of sand fleas and excluded from the study for ethical reasons; however, this procedure was not required during the trial. All lesions were classified, quantified, photographed, and documented in the parasitological skin examination records.

**Table 1:** Severity score for acute clinical signs of designated topographic tungiasis

Clinical signs	Number of locations affected	Score
Hyperemia and/or edema^a^	1–5	1
	6–10	2
	11–16	3
Pain on digital pressure	1–5	1
	6–10	2
	11–16	3
Suppuration and/or formation of abscesses^a^	1–5	1
	6–10	2
	11–16	3
Clustering of lesions ^b^	1–5	1
	6–10	2
	11–16	3
Fissure(s) ^a^	1–5	1
	6–10	2
	11–16	3
Skin ulceration	1–5	1
	6–10	2
	11–16	3
Mutilation of lesions regardless of the sites involved ^c^		2
Altered gait/lameness		3
Ectopy of lesions		0.5 ^d^

^a^Regardless of the number of foci and the size of the area involved

^b^Three or more lesions close together (1–2 mm apart)

^c^Mutilation of lesions indicates severe itching

^d^For each ectopic body part involved, up to a maximum of eight ectopic sites; maximum four points

Therefore, the maximum individual score (SCADT) for a dog was 27 (23 + 4)

### Statistical analysis

Statistical analysis was performed to assess the treatment efficacy using R version 3.6.1 software, with dogs as the experimental unit. The primary efficacy assessment of the product was based on the percentage of dogs free of live fleas (stages II and III) in the treatment group. The 95.0% confidence limits for the percentage of dogs free of live *T. penetrans* were calculated as Wilson scoring intervals. At each time point of post treatment evaluation, Fisher’s exact test (unilateral, using Casagrande, Pike, and Smith [27] continuity correction) was used with a significance level of *α* = 0.025 to compare the percentage of dogs free of parasites between the TG and the CG. The resulting *P* values, odds ratios (OR), and 95.0% confidence limits for the OR were obtained from the PROC FREQ using the Taylor series approach.

Secondary efficacy was calculated according to the number of live fleas (stages II and III) in each group on each evaluation day by calculating geometric and arithmetic means using the following formula:

$${\text{Secondary efficacy}} \left( \% \right) = 100 \times \frac{{ \left( {{\text{Dc at day X}} {-} {\text{Dt at day X}}} \right)}}{{\text{Dc at day X}}}$$

where *Dc* is the mean number of live fleas (geometric and arithmetic) in the control group (total lesions/number of animals), *Dt* is the mean number of live fleas (geometric and arithmetic) in the treated group (total lesions/number of animals), and *X* is the experimental day.

Notably, the geometric mean is used in cases of counting zero fleas on a dog, according to the following equation:

$$Geometric \,mean \left[ {X_{g} } \right] = \left( {\mathop \prod \limits_{i = 1}^{n} \left( {X + 1} \right)} \right)^{\frac{1}{n}} - 1$$

where *Xg* is the geometric mean and *n* is the number of individuals in the group.

To be considered effective, a flea product must reduce parasites by more than 90.0% [28]; therefore, this percentage was used in this study to assess formulation effectiveness.

Arithmetic mean and mean SCADT scores in the two groups were compared at each post-treatment evaluation timepoint using Shapiro–Wilk test to identify the normality of data. If the data distribution was nonnormal, the Mann–Whitney *U* test was performed with the level of significance set to *α* = 0.05 (two-sided).

## Results

The study dogs, aged between 3 months and 11 years, were all mixed breed, intact, and weighed between 2.5 and 33.3 kg. On day 0, before treatment, *T. penetrans* mean live flea counts were 10.3 ± 9.8 in treated dogs and 11.5 ± 13.9 in untreated dogs, whereas the flea count per dog was 1–94 fleas on treated dogs and 1–108 on control dogs. The study groups were comparable on day zero in terms of age, weight, sex, and SCADT distribution. The data from the animals in each group are presented in Table 2.

**Table 2:** Age, weight, sex, breed, flea count, and SCADT on day 0

	Treated group	Group control
Number of dogs (*n*)	32	32
Age (years) (mean ± SD)	3.4 ± 2.4	3.9 ± 3.1
Age (median)	3	3
Age range	3 months to 10 years	3 months to 11 years
Body weight (kg) (mean ± SD)	9 ± 4.7	9.4 ± 6.8
Body weight (median)	8.1	7.7
Body weight range (kg)	3–19.7	2.5–33.3
Intact females	14 (43.2%)	15 (46.8%)
Intact males	18 (56.2%)	17 (53.1%)
Mixed breed	32 (100.0%)	32 (100.0%)
Flea count (mean ± SD)	10.3 ± 9.8	11.5 ± 13.9
Flea count (median)	7.5	6
Flea count range	1–94	1–108
SCADT (mean ± SD)	3.0 ± 2.6	2.3 ± 2.2
SCADT (median)	2.5	2.0
SCADT range	0–14	0–9

*SD* standard deviation

Three dogs died during the study, but none of the deaths were related to the administered treatment. One dog from the control group died due to the ingestion of a toxic substance applied at home by the owner, and the other two animals (one from the control group and one from the treated group) were hit by cars. However, data collected until the time of their deaths were considered in the analyses. No adverse clinical effects such as systemic allergic responses, vomiting, or diarrhea were reported.

### Primary efficacy

**Fig. 1:**
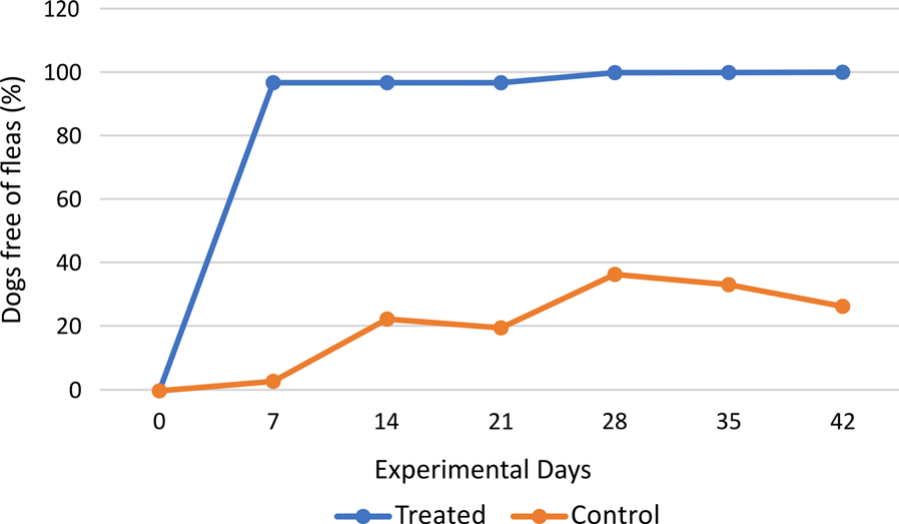
Percentage of flea-free dogs on each assessment day by study group

Data related to primary efficacy (calculated based on the number of animals without fleas) are shown in Table 3 and illustrated in Fig. 1. From days 7–21, the primary efficacy remained above 95.0%, reaching 100.0% (31/31) between days 28 and 42. In the CG, the proportion of parasite-free animals did not exceed 40.0% throughout the study period. Statistical comparison of parasite-free individuals between the TG and CG demonstrated that treatment resulted in significant differences between the groups on each experimental day throughout the study from day 0 to 42 (*P* < 0.0001, statistical values of each evaluation day are in Table 3). Therefore, the treatment was effective in preventing *T. penetrans* infestation in dogs.

**Table 3:** Number and percentage of flea-free dogs by study day and study group

Day	Treated group		Group control		*P* value ^b^(one-sided)	OR
	Free from fleas (*n*)	Percentage (%)	Free from fleas (*n*)	Percentage (%)		OR ^c^ [95% CI^a^]
7	31 (32)	96.9 (84.2–99.4)	1 (31)	3.2 (5.5–15.7)	< 0.0001	930.0 [55.1; 15,554.1]
14	31 (32)	96.9 (84.2–99.4)	7 (31)	22.6 (11.4–39.8)	< 0.0001	106.3 [12.2; 923.5]
21	31 (32)	96.9 (84.2–99.4)	7 (30)	20.0 (11.7–40.9)	< 0.0001	101.7 [11.1; 886.4]
28	31 (31)	100.0 (88.9–100.0)	11 (30)	36.7 (21.8–54.4)	< 0.0001	50.5 [6.4; 448.4]
35	31 (31)	100.0 (88.9–100.0)	10 (30)	33.3 (19.2–51.2)	< 0.0001	52.0 [7.4; 552.3]
42	31 (31)	100.0 (88.9–100.0)	8 (30)	26.7 (14.1–44.5)	< 0.0001	85.2 [9.9; 731.5]

^**a**^*CI* confidence interval

^**b**^Fisher’s exact test, *α* = 0.025

^**c**^*OR* Odds ratio, *CI* Confidence Interval

### Secondary efficacy

The secondary efficacy (based on the number of live fleas) of fluralaner on day 7 was 99.7%, remaining above 99.0% from days 7 to 21, and reaching 100.0% from days 28 to 42. The arithmetic and geometric means were similar on all experimental days in the treated group. The arithmetic mean of live fleas was significantly low in the treated dogs (*P* < 0.0001, statistical values of each evaluation day are in Table 4) from days 7 to 42. The arithmetic means of live fleas in the CG varied between 3.7 and 7.1 and in the TG varied between 0.0 and 0.4, during all the experimental days of the study.

**Table 4:** *Tunga penetrans* counts (percentage of efficacy and *P*-values) in treated and untreated dogs on all treatment days

Day	Geometric mean live flea counts			Arithmetic mean live flea counts			*P* value	Statistical value (*W*) W
	TG	CG	Efficacy %	TG	CG	Efficacy %		
0	6.84	7.07	–	10.34	11.56	–	0.839	472.5
7	0.01	4.08	99.7	0.01	6.17	99.8	< 0.0001	975
14	0.02	4.00	99.5	0.03	5.00	99.7	< 0.0001	874.5
21	0.03	4.86	99.4	0.04	7.10	99.4	< 0.0001	856
28	0.00	6.87	100.0	0.00	5.00	100.0	< 0.0001	759.5
35	0.00	5.40	100.0	0.00	4.50	100.0	< 0.0001	775
42	0.00	3.52	100.0	0.00	3.70	100.0	< 0.0001	821.5

Wilcoxon test (Mann–Whitney *U* test), *α* = 0.05

**Fig. 2:**
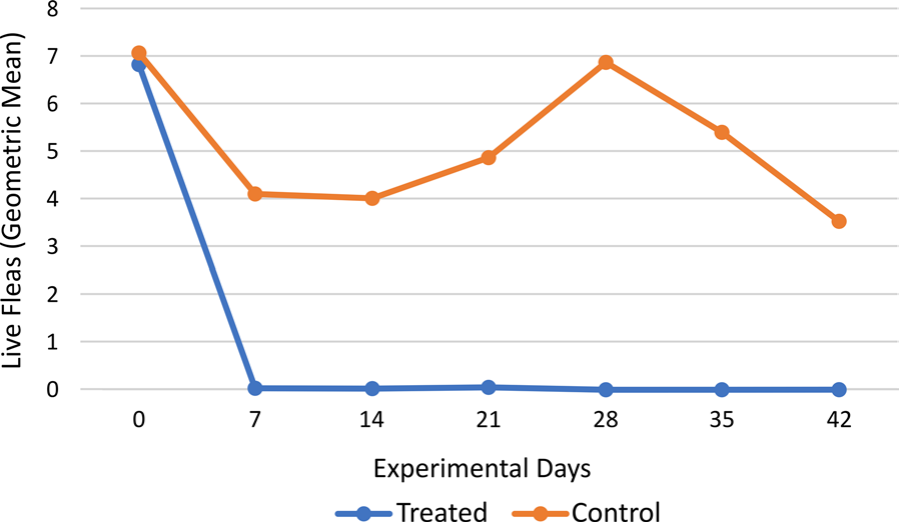
Geometric means of live flea counts by study group over time

The geometric means of live flea counts for each study group at all evaluation times are shown in Fig. 2.

**Fig. 3:**
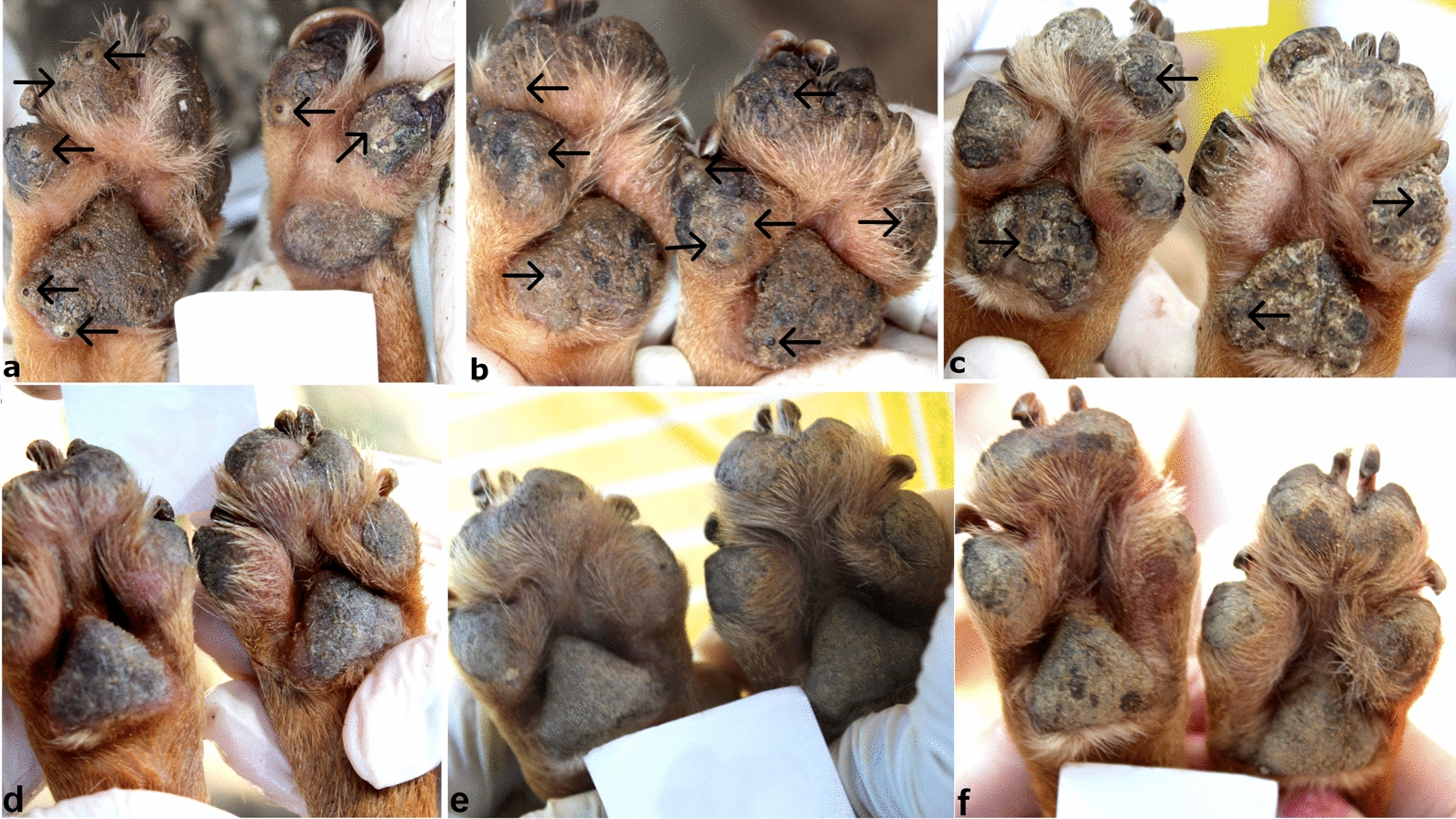
Evolution of the lesions through the study for dog 36 of the TG. **a**, **b** On day 0, the dog had multiple lesions caused by *T. penetrans* in stages II and III located on the pads (arrows). **c** On day 7, after treatment, the footpads no longer present any vital lesions; it is possible to observe lesions in stages IV and V in the anterior and posterior paws (arrows). **d** On day 21, the pads still remain without vital lesions. **e**, **f** On the 35th and 42th day of evaluation, respectively, the pads were free of *T. penetrans* lesions and completely re-epithelialized. Source: Personal archive

**Fig. 4:**
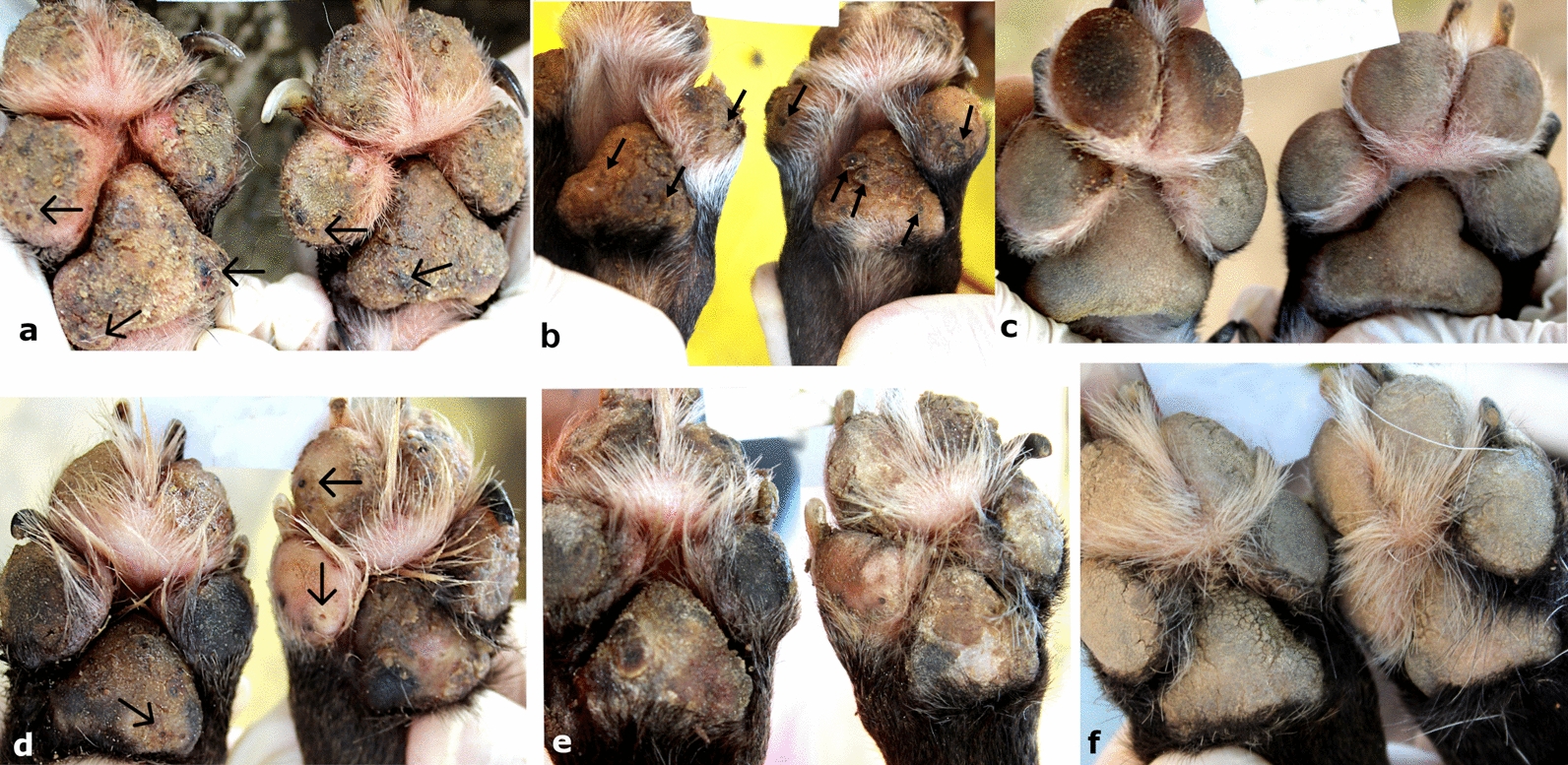
Evolution of vital lesions in two treated dogs. **a** Animal 12 of the TG on day 0, with multiple lesions in stages II and III in the forepaws (arrows). **b** On day 7 of the evaluation, dog 12 after treatment, the footpads no longer present any vital lesions, it is possible to observe lesions in stages IV and V on the paws (arrows). **c** The same dog 12 on day 42 post-treatment with completely healthy front paws and no lesions caused by *T. penetrans*. **d** Animal 24 of the TG on day 0 with several vital lesions on the hind paws (arrows); **e** Animal 24 on day 7 of the study showing hind paws with desquamated epithelium, in the phase of re-epithelialization. **f** The same dog on day 42 of evaluation without the presence of lesions or scars from lesions caused by *T. penetrans* on the hind paws. Source: Personal archive

**Fig. 5:**
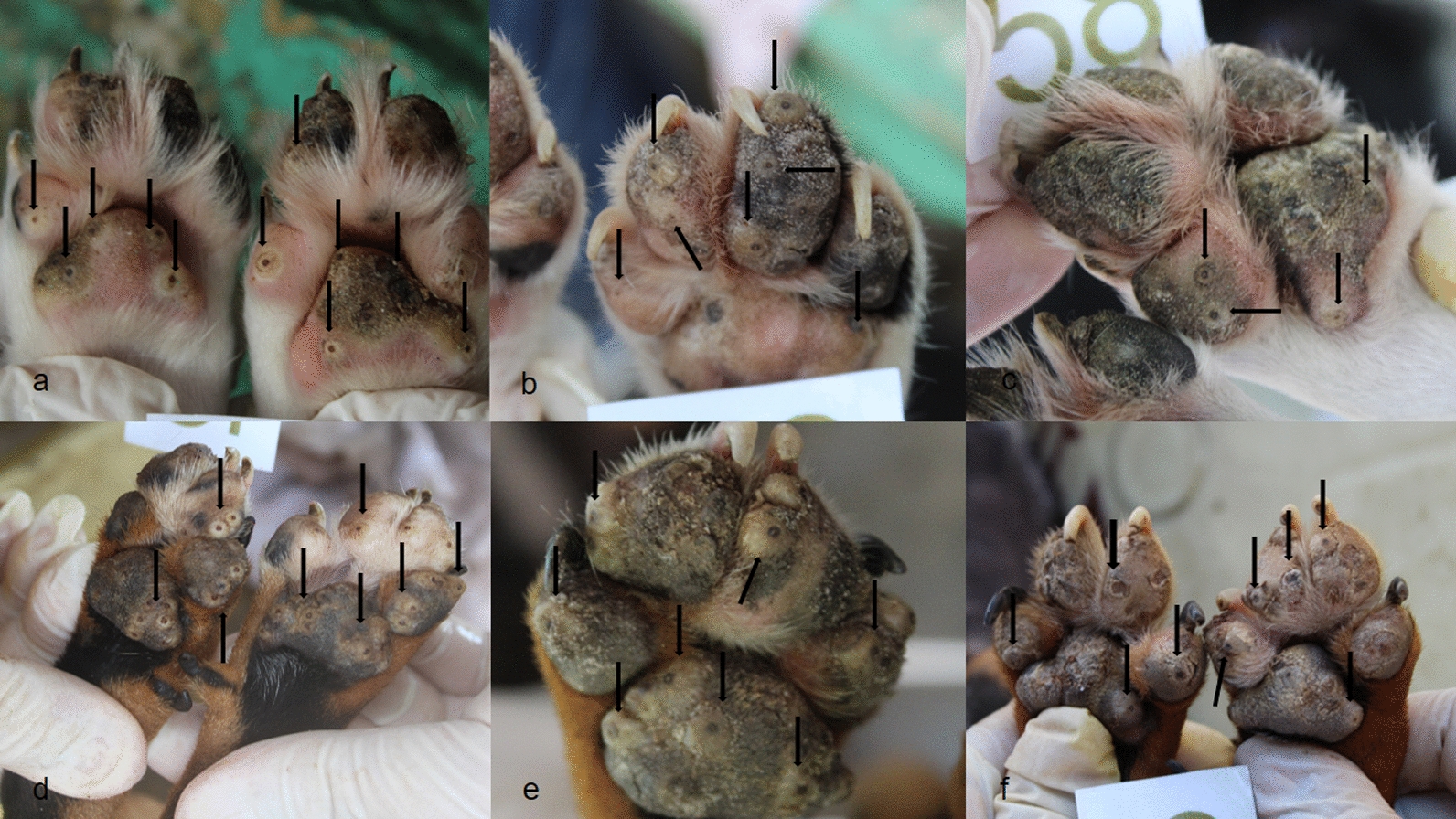
Evolution of vital lesions in two untreated dogs (arrows). **a**–**c** Animal 58 from the CG in evaluation days 0, 7, and 42, respectively, with stages II and III on forepaws. **d**–**f** Animal 46 of the CG, on evaluation days 0, 7, and 42 with several vital lesions (II and III) on the front paws. Source: Personal archive

The presence of lesions before and after treatment was monitored using the photographic records of the enrolled dogs. Examples of these three animals are shown in Figs. 3, 4 and 5.

### Total SCADT

The means of the SCADT by study day and study group as well as the amplitude ranges are shown in Table 5. The mean SCADT score differed significantly between the groups from days 7 to 42 (*P* < 0 0.05, statistical values of each evaluation day are in Table 5). On days 35 and 42, the mean severity scores in the treatment group were zero.

**Table 5:** SCADT by study day and study group

Day	Treated		Control		*P* value	Statistical value (*W*)
	Mean ± SD	Median	Mean ± SD	Median	(two-sided)	
0	2.90 ± 2.50	2.5	2.30 ± 2.20	2	0.15	634
7	0.84 ± 1.03	0	2.47 ± 2.15	2	0.00	259
14	1.59 ± 1.76	1.50	2.60 ± 1.94	2	0.04	335
21	0.62 ± 1.49	0	2.54 ± 2.31	2.5	0.00	226
28	0.14 ± 0.56	0	1.50 ± 1.80	1	0.00	234.5
35	0.00 ± 0.00	0	1.28 ± 1.40	1	0.00	108
42	0.00 ± 0.00	0	1.00 ± 1.36	0	0.00	246.5

Wilcoxon test (Mann–Whitney *U* test), *α* = 0.05

## Discussion

The Bravecto^®^ 1-Month (Defenza^®^) formulation, with monthly indication, was chosen for the study due to its potent systemic active compound from the isoxazoline family with acaricidal and insecticidal action. The formulation is effective against infestations caused by several species of ectoparasites [29–31], including *T. penetrans* fleas [23], and has a lower cost than that of Bravecto^®^ chewable tablet, which is effective for 3 months.

Fluralaner is a potent inhibitor of the GABA-controlled chloride channel with specific action on arthropods, consequently causing the death of the ectoparasite [32]. The systemic action of fluralaner is an important pharmacological characteristic because its effect is not affected by rain or bathing, which represents an advantage when compared to topical products with the same pharmacological action [33, 34]. This particularity is relevant for dogs raised in semi-domesticated areas, as they are exposed to rainwater and swim in rivers and seas, as was the case for the animals in this study.

We observed that a single oral administration of fluralaner at a dose of 10–18 mg/kg (Bravecto^®^ 1-Month/Defenza^®^) effectively protected dogs against *T. penetrans* infestation for at least 6 weeks under field conditions in an endemic region with high infestation rates (i.e., high environmental exposure). In the first 3 weeks after treatment, the primary efficacy was greater than 95.0%, reaching 100.0% protection in the 4th week and remaining for the following 2 weeks. Notably, this is one of the first monthly formulations tested with proven efficacy against *T. penetrans* in dogs. Considering that the monthly formulation of fluralaner presented similar results to those achieved by the quarterly formulation for the treatment of canine tungiasis [23], we inferred that even at a low dosage, the effectiveness of the active principle in the monthly formulation would remain high.

Additionally, secondary efficacy ranged from 99.0% to 100.0%, thereby revealing the treatment’s impact on the *T. penetrans* reproductive cycle, which was previously demonstrated in a similar article [23]. The reduction in the number of flea-releasing eggs in the environment may favor the reduction of infestation in animals that share the same environment as the treated dogs. This environmental effect is a possible cause for the reduction in the number of tungiasis-positive dogs in the untreated group [35].

Furthermore, the low SCADT averages observed in the treated animals demonstrated that they showed few acute local clinical signs such as pain, edema, hyperemia, and ulceration, compared with untreated dogs, as observed in similar studies [23, 35]. In view of this, the present results allow us to infer that the treatment provides an improved quality of life and consequently improves the well-being of animals by eliminating sand fleas. Furthermore, no dog treated with Bravecto^®^ 1-Month (Defenza^®^) manifested any adverse reactions after administration, demonstrating that the formulation is safe for dogs [23, 36–38].

The formulation is effective in combating *T. penetrans* for at least 42 days; hence, we also suggest the formulation’s use as a disease control tool for owners who wish to medicate their dogs for a short period of time. This will aid in treating or preventing infestations, especially in the case of tourists traveling to tungiasis-endemic areas, such as the area in this study. We emphasize that the control of tungiasis is a public health issue, especially in endemic areas of Brazil, where dogs are identified as one of the main risk factors for tungiasis.

We suggest the potential use of Bravecto^®^ 1-Month (Defenza^®^) in public health programs aimed at controlling tungiasis, as well as in endemic areas by dog owners. This is owing to the formulation’s commercial availability and low cost compared with that of other proven effective options.

## Conclusions

The Bravecto^®^ 1-Month (Defenza^®^) formulation, orally administered in a single dose of 10–18 mg/kg, was effective against natural *T. penetrans* flea infestations in dogs 7–42 days after administration. The dogs treated in this study showed an improvement in SCADT associated with tungiasis.

## Data Availability

The authors confirm that the data supporting the findings of this study are available within the article.
